# A hierarchical model of social perception: Psychophysical evidence suggests late rather than early integration of visual information from facial expression and body posture

**DOI:** 10.1016/j.cognition.2018.12.012

**Published:** 2019-04

**Authors:** Christoph Teufel, Meryl F. Westlake, Paul C. Fletcher, Elisabeth von dem Hagen

**Affiliations:** a Cardiff University Brain Research Imaging Centre, School of Psychology, Cardiff University, UK; b Department of Psychiatry, University of Cambridge and Cambridgeshire and Peterborough NHS Foundation Trust, UK

**Keywords:** Social perception, Facial expression, Aftereffect, Adaptation, Body posture, Perceptual integration

## Abstract

Facial expressions are one of the most important sources of information about another’s emotional states. More recently, other cues such as body posture have been shown to influence how facial expressions are perceived. It has been argued that this biasing effect is underpinned by an early integration of visual information from facial expression and body posture. Here, we replicate this biasing effect, but, using a psychophysical procedure, show that adaptation to facial expressions is unaffected by body context. The integration of face and body information therefore occurs downstream of the sites of adaptation, known to be localised in high-level visual areas of the temporal lobe. Contrary to previous research, our findings thus provide direct evidence for late integration of information from facial expression and body posture. They are consistent with a hierarchical model of social perception, in which social signals from different sources are initially processed independently and in parallel by specialised visual mechanisms. Integration of these different inputs in later stages of the visual system then supports the emergence of the integrated whole-person percept that is consciously experienced.

## 1 Introduction

Facial expressions provide important information about a person’s emotional state (Bruce & Young, 2012). Conventionally, research has focussed on how observers extract information about emotions from faces alone. However, in our everyday lives, we typically encounter faces together with a body. More recently, several studies have therefore highlighted the importance of body context in the processing of facial expression: a body posture showing an incongruent emotion biases perception of the facial emotion towards that of the body (for reviews, see de Gelder et al., 2006, Hassin et al., 2013). For instance, Aviezer et al. (2008) showed that facial expressions of disgust were often categorized as anger when presented in the context of an angry body.

The biasing influence of body posture on categorization of facial expressions is thought to be genuinely perceptual, rather than arising from post-perceptual interpretative processes (Aviezer, Trope, & Todorov, 2012). Indeed, it has been suggested that the integration of information from face and body underlying these biases occurs early in the visual system (Aviezer et al., 2008, Aviezer et al., 2011, Meeren et al., 2005). Potential candidate substrates have been suggested to be localized in lateral and dorsal occipital cortex (Meeren et al., 2005), e.g., the occipital face area and the extrastriate body area. However, current models of face processing provide substantial evidence to suggest that representations of facial expressions are underpinned by processes in areas further downstream, in particular superior temporal sulcus (STS) and, to some extent, fusiform face area (FFA) (e.g., Duchaine and Yovel, 2015, Haxby et al., 2000). Integration of emotional face and body information in occipital areas would therefore suggest that facial expression information would have to be extracted much earlier in the processing stream than current evidence suggests.

One powerful approach from visual psychophysics to exploring how faces are coded by the brain capitalises on perceptual aftereffects resulting from adaptation (Webster & MacLeod, 2011). Notably, specific and selective aftereffects in the perception of facial expression have been demonstrated (e.g., Butler et al., 2008, Cook et al., 2011, Fox and Barton, 2007, Hsu and Young, 2004, Rhodes et al., 2017, Skinner and Benton, 2010, Webster et al., 2004, Xu et al., 2008). For example, Webster et al. (2004) showed that after extended exposure to disgusted facial expressions, ambiguous faces that were generated by morphing between disgust and surprise were perceived as looking more surprised, i.e., perception was biased away from the adaptor expression. Such aftereffects are thought to occur because of a re-calibration process during which neurons tuned to the adapting expression selectively change their response properties with extended exposure. Several neuronal mechanisms potentially contribute to these changes in responsiveness (Grill-Spector, Henson, & Martin, 2006). As a consequence of the neuronal changes, when presented with an ambiguous target face, adapted neurons contribute less to perception relative to non-adapted neurons, leading to a specific and measurable perceptual bias away from the adaptor expression (see discussion and, for a review, Webster & MacLeod, 2011).

Adaptation to facial expressions occurs at different levels of the visual information-processing hierarchy (Xu et al., 2008). However, several manipulations indicate that it is predominantly a high-level, configural phenomenon (Webster & MacLeod, 2011). For example, facial expression aftereffects are robust to changes in size (Hsu & Young, 2004) and position (Skinner & Benton, 2010) between adaptor and target stimulus, and are also observed under free viewing conditions (Butler et al., 2008), all of which are inconsistent with adaptation to low-level stimulus features. Furthermore, facial expression aftereffects are also relatively insensitive to changes in facial identity between adaptor and target stimulus (Fox & Barton, 2007). Together, these findings suggests that facial expression adaptation paradigms mainly target high-level face representations, rather than low-level features (Webster & MacLeod, 2011). Dovetailing with these psychophysical findings, human neuroimaging studies and intracranial recordings indicate that extended exposure to facial characteristics such as expressions (e.g., Fox et al., 2009, Furl et al., 2007, Furl et al., 2007, Ganel et al., 2005, Winston et al., 2004) and identity (e.g., Gilaie-Dotan and Malach, 2007, Ng et al., 2006, Quian Quiroga et al., 2014, Rotshtein et al., 2005) mainly, but not exclusively, affects neuronal populations within high-level visual areas of the temporal lobe, including STS and FFA.

In this study, we examined the influence of body context on perceptual categorization of, and adaptation to, facial expressions. If face and body signals are integrated prior to the processing stages underlying facial expression aftereffects, extended exposure to congruent vs. incongruent face-body stimuli should lead to aftereffects that shift perception in opposite directions. Viewing a disgusted face in an angry body context should target neuronal processes tuned to angry expressions more and those tuned to disgust less than exposure to the physically identical, but perceptually distinct disgusted expression in a disgusted body context. More simply, if a disgust face is perceived as disgusted then the adaptation paradigm should result in a perceptual shift such that ensuing morphed faces will be seen as angry. If however, the disgust face is perceived as angry because of an angry body context, then adaptation to anger should occur. Crucially, this happens only if contextual integration occurs prior to the sites of adaptation, such that categorization and adaptation rely on similar whole-person representations. If face and body are integrated after the processing stage targeted by the adaptation paradigm, exposure to a disgusted face should lead to the same aftereffects, regardless of any context-induced categorization bias. Whether adaptation is context-sensitive can thus help pinpoint the level at which face and body signals are integrated along the visual processing hierarchy. It effectively provides an index to determine the upper or lower bound of integration. Furthermore, it provides insights into the functional role of adaptation to high-level stimulus properties such as facial expressions by uncovering the extent to which changes in response properties of the adapted mechanisms are a response to the isolated physical aspects of the stimulus or the integrated percept that is consciously experienced.

## 2 Overview of experiments

We conducted seven experiments, across four studies. Study 1 showed a robust biasing effect of body context on categorization of disgusted facial expressions in an experiment similar to previous studies (e.g., Aviezer et al., 2008, Aviezer et al., 2011) but no influence on aftereffects; Study 2 maximised the effect size in the categorization experiment but, again, showed no effect of body on aftereffects; Study 3 combined the categorization and adaptation paradigms into one single experiment, replicating findings of the previous two studies; finally, Study 4 established similar findings with a different facial expression.

## 3 Study 1: Body context influences perceptual categorization but not adaptation

Study 1 aimed to replicate the biasing effect of body context on perceptual categorization of facial expressions, and to assess, using an adaptation paradigm, whether these biases result from an early integration of information from face and body.

### 3.1 Method

#### 3.1.1 General outline

The extent to which body posture impacts on perceptual judgments of facial expressions is strongly influenced by the physical similarity of the facial characteristics of congruent and the incongruent emotions (Aviezer et al., 2008). To maximise effects, we therefore focussed on facial expressions of disgust and anger.

All observers had normal or corrected-to-normal visual acuity and were native English speakers. Experimental protocols for all experiments were approved by the local ethics committee and are in line with the Declaration of Helsinki. Written informed consent was obtained prior to participation. For all experiments, we chose sample sizes based on effect sizes found in in-lab piloting studies. The sample size is comparable to those used in other studies on categorization of, and adaptation to, facial expressions.

#### 3.1.2 Observers

A total of 24 naïve observers (6 male; age in years (mean ± std): 22.1 ± 3.4) participated in Study 1. One observer was excluded from the analysis due to computer failure during data acquisition on one day. In the adaptation experiment, two more participants were excluded from the analysis because of a poor fit between data and psychometric function in one or more conditions as indicated by the goodness-of-fit analysis.

#### 3.1.3 Apparatus

The same apparatus was used for all 4 studies. Stimuli were presented on a Samsung SyncMaster 2233RZ monitor, driven by a PC computer at a refresh rate of 60 Hz and a spatial resolution of 1680 × 1050 pixel with 8-bit grayscale resolution. In a dimly-lit room, observers used a chin rest to maintain a constant viewing distance of 55 cm. Stimuli were presented using the Psychophysics Toolbox (Brainard, 1997, Kleiner et al., 2007) in Matlab R2015a (The MathWorks, Inc., Natick, MA, USA).

#### 3.1.4 Stimuli

**Fig. 1:**
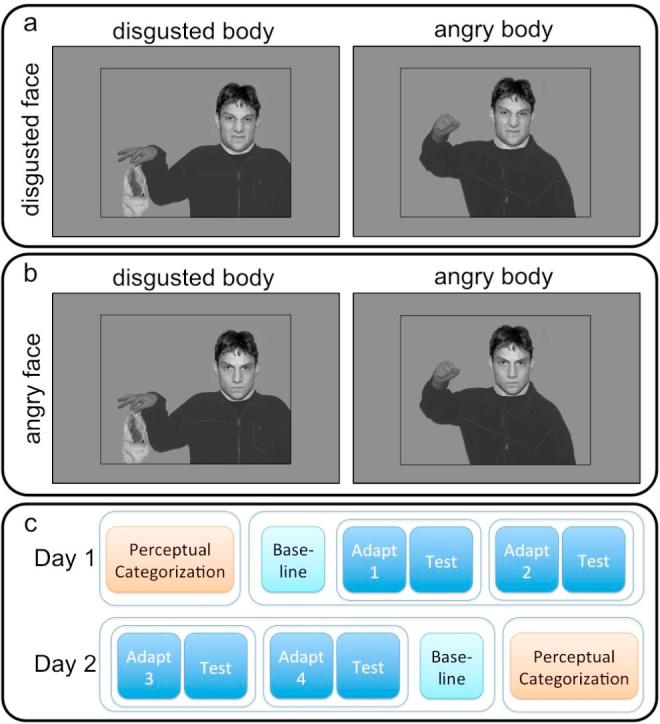
Illustration of the compound face-body stimuli used in Studies 1 to 4 and the protocol. (a) In the Perceptual Categorization experiment of Study 1 and 2, disgusted faces of different facial identities were shown in a disgusted and angry body context as illustrated here (as well as in a fearful and sad body context, not shown). For the Adaptation experiment, the same face-body stimuli were used as adaptors. In Study 1, observers were also adapted to the body context alone with faces blurred (not illustrated here). (b) In Study 4, angry faces in angry and disgusted contexts served as stimuli for the Perceptual Categorization experiment and as adaptors in the Adaptation experiment. (c) Protocol of Study 1. The protocol of studies 2 and 4 was identical except that only one adaptation and test session was conducted. Study 3 used a one-day protocol.

Face images from two validated databases of facial expressions – the NimStim Set of Facial Expressions (Tottenham et al., 2009) and the Radboud Faces Database (Langner et al., 2010) – were used to generate the stimuli. In the Perceptual Categorization experiment, we used 24 grayscale images of different male Caucasian and African-American identities with disgusted and angry facial expressions. Faces were stripped of the background using Adobe Photoshop 6.0 (Adobe Systems, San Jose, CA, USA) and were merged with bodies expressing disgust, fear, sadness, or anger to produce both congruent and incongruent face-body stimuli (Fig. 1a,b); the images of the bodies were obtained from a previous study (Aviezer et al., 2008). The whole stimulus, including body parts and face, subtended roughly 12° of visual angle horizontally and 12° vertically; faces subtended approximately 3.5° horizontally and 5.7° degrees vertically. All stimuli were presented on a mean grey background.

In the Adaptation experiment, we used the face-body stimuli showing a disgusted expression in a disgusted or angry body context as adaptation stimuli. Additionally, two further stimuli were used, where the faces were blurred with a cosine window so that no facial expression (or facial identity) was visible. Thus, a disgusted or angry body context on its own was used as adaptor.

Target probes were generated from grayscale images of two different male Caucasian identities. Using the Fantamorph Professional software (Abrosoft Co.), morphs between a fully angry and a fully disgusted facial expression were created for both identities. Targets were presented through an oval window with cosinusoidally modulated edges, the unmodulated part of which subtended approximately 6° of visual angle horizontally by 8.5° vertically, cropping hair, ears, and neck so that only the internal facial features were visible.

In the current study, we specifically wanted to target those neuronal populations that encode high-level visual representations of facial expressions, avoiding contamination of the results by adaptation to low-level features. We therefore presented adaptor and target at different spatial scales, at different positions, and used a number of different identities for adaptation under free viewing.

#### 3.1.5 Procedure

Study 1 was conducted on two separate testing days: Each day comprised a session of the Perceptual Categorization experiment, an assessment of baseline performance for the Adaptation experiment, and two experimental sets of the Adaptation experiment (Fig. 1c). On each day, one set of the Adaptation experiment always included a condition with a face, the other without. The order of these was counterbalanced and they were separated by at least 10 min of rest. The type of body context shown on the first vs. the second day was counterbalanced. The Perceptual Categorization sessions were identical on both days and were conducted at the start of day one and at the end of day two to ensure that extensive viewing of the compound face-body stimuli during adaptation did not affect the Perceptual Categorization task.

***Perceptual Categorization.*** Observers categorized the facial expressions of compound face-body stimuli (while explicitly instructed to ignore the body) as disgusted, angry, fearful, or sad in four different conditions. Although we were particularly interested in categorization of disgusted faces presented in the context of a disgusted or an angry body, to ensure our design was similar to previous studies (Aviezer et al., 2008; 2012; Noh & Isaacowitz, 2013), the disgusted face was also shown in the context of a fearful and a sad body context.

**Fig. 2:**
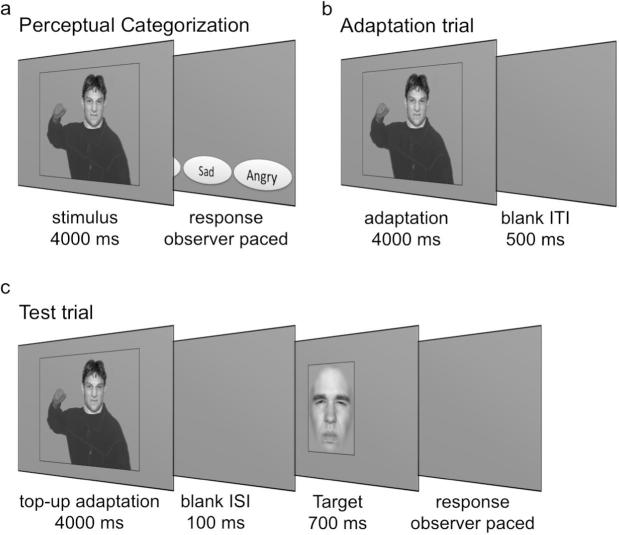
Illustration of the experimental designs of Study 1. (a) Perceptual Categorization experiment. Observers were shown a face-body stimulus and were asked to indicate the facial expression by clicking on one of four buttons (buttons are not to size). (b) During adaptation observers were adapted to one of four face-body combinations: a disgusted face was shown in an angry – as illustrated here – or a disgusted body context; in the remaining conditions, the body contexts were shown with a blurred face so that no facial expression was discernable. Test trials (c) consisted of a top-up adaptation stimulus followed by a target facial expression (morph between disgusted to angry). All indicated timings include a sinusoidally modulated ramp into (100 ms) and out of the full image (100 ms). This was used to minimize retinal aftereffects.

Within each session, each of the 24 facial identities was shown in each of the four body contexts, resulting in a total of 96 trials per session. The sequence of trials was pseudo-randomized such that the same identity was never shown in consecutive trials. In every trial, a face-body stimulus was followed by presentation of four response-option buttons, that could be selected via mouse click (Fig. 2a). To avoid any motor biases, the pairings of the button location to the emotion label were randomly assigned on each trial. Responses were observer-paced and followed by a 0.5 ms intertrial interval.

***Adaptation.*** The Adaptation task was presented in four different conditions that were identical except for the nature of the adaptation stimuli. In two conditions, these showed a disgusted facial expression in the context of either a disgusted or angry body posture. The physical properties of the facial expressions in these conditions were identical but we were interested in whether the body contexts would have an influence on adaptation. In order to exclude the possibility that potential differences in adaptation in these conditions are due to effects of the body independently of the face, we also included two further conditions, in which observers were adapted to body contexts without a face. Each of these conditions was conducted in an experimental block, consisting of 144 test trials presented after observers had been exposed to 24 adaptation trials.

In each adaptation trial (Fig. 2b), observers binocularly free-viewed a stimulus that consisted of a face showing disgust in the context of a body expressing either disgust or anger (Fig. 1a), or just the body context without a face. In one third of the trials, a red dot appeared 200 ms after stimulus onset and was shown for 200 ms in one of eight locations on the person depicted in the image. Observers were instructed to look carefully at the stimuli and indicate detection of the dot with a response button. The purpose of this sham manipulation was to ensure careful viewing of the adaptation stimuli. The 24 different facial identities used to generate the adaptation stimuli were shown in randomized order.

A 2-alternative-forced-choice (2AFC) paradigm was used to present test trials. Each test trial consisted of a top-up adaptation stimulus followed by a blank screen, and a target stimulus (Fig. 2c). The identities used for the top-up adaptation stimulus were shown in randomized order. A morphed face showing an ambiguous expression between anger and disgust served as target. The morph level was determined by the the Psi method (Kontsevich & Tyler, 1999), an adaptive procedure, which chooses stimulus levels in such a way as to minimise expected entropy regarding point of subjective equality (PSE) and slope value of the psychometric function. Observers were asked to indicate whether the test stimulus showed an angry or a disgusted expression by pressing the respective response button. The response period was observer-paced and was followed by a blank intertrial interval. On each testing day, baseline testing was conducted, consisting of 144 trials each. Baseline trials were similar to test trials but omitted the top-up adaptation stimulus.

#### 3.1.6 Data analysis

Statistical analyses were performed with the software R (The R Foundation for Statistical Computing). To confirm that null effects were due to the absence of an effect rather than the absence of evidence for an effect, we conducted an additional analysis for such data with a Bayesian procedure using the software JASP (JASP Team 2016; jasp-stats.org). Effect sizes were calculated using the Measures of Effect Size toolbox implemented in Matlab (Hentschke & Stüttgen, 2011)

***Perceptual Categorization.*** The main focus of our analysis is on the influence of disgusted and angry body contexts on categorization of disgusted faces. For completeness, we report individual comparisons and uncorrected p-values. However, all reported p-values would survive Bonferroni correction within experiments, i.e., four comparisons for the Categorization tasks and three for the Adaptation tasks. Importantly, however, note that this type of correction is conservative given that not all tests were necessarily independent.

To relate performance in the Categorization and the Adaptation experiment, we used two different Bias Indices (equation (1), (2)). For both indices, we only considered disgust and anger responses, and only disgust and anger body contexts. For Bias Index 1, we calculated the proportion of correct responses in the Categorization task relative to all disgust and anger responses for congruent and incongruent body contexts. For instance, in Study 1, in which a disgusted face was used, the disgust body posture was the congruent context and the angry posture the incongruent context. The index was then derived by forming the ratio of these proportions:

(1) $$\frac{n_{\mathrm{corrResp}}^{\mathrm{conContext}}}{\left(n_{\mathrm{disResp}}^{\mathrm{conContext}}+n_{\mathrm{angResp}}^{\mathrm{conContext}}\right)}/\frac{n_{\mathrm{corrResp}}^{\mathrm{inconContext}}}{\left(n_{\mathrm{disResp}}^{\mathrm{inconContext}}+n_{\mathrm{angResp}}^{\mathrm{inconContext}}\right)}$$

where n is the number of responses, with the superscript indicating the body context as congruent (conContext) or incongruent (inconContext). The subscript indicates the type of response as either correct (corrResp), disgust (disResp) or anger (angResp). For instance, in Experiment 1, in which disgusted faces were shown, $n_{\mathrm{corrResp}}^{\mathrm{conContext}}$ is the number of disgust responses in the disgust body context. This Bias Index provides a measure of the proportional change in disgust and anger categorizations across disgust and anger body contexts. It increases with more anger, and less disgust categorizations in the anger body context compared to the disgust body context.

Bias Index 1 is intuitively meaningful but, due to it being a ratio, has the disadvantage that potential outliers could skew the analysis. We therefore calculated a second index, Bias Index 2, based on d’ from signal detection theory (Macmillan & Creelman, 2005):

(2) $$z\left(\frac{n_{\mathrm{corrResp}}^{\mathrm{conContext}}}{\left(n_{\mathrm{disResp}}^{\mathrm{conContext}}+n_{\mathrm{angResp}}^{\mathrm{conContext}}\right)}\right)-z(\frac{n_{\mathrm{corrResp}}^{\mathrm{inconContext}}}{\left(n_{\mathrm{disResp}}^{\mathrm{inconContext}}+n_{\mathrm{angResp}}^{\mathrm{inconContext}}\right)}$$

where z indicates the standard score. In case the proportion of responses was at the extreme of 0 or 1, values were adjusted by assigning 1/N or 1–1/N to these proportions, respectively (Macmillan & Creelman, 2005).

Both indices do not take into account fear or sadness categorizations as simpler measures such as the ratio of disgust (or anger) responses in the two contexts would. However, the advantage of the chosen indices is that they are sensitive to changes in both disgust *and* anger responses at the same time. Importantly, note that changes in fear and sadness categorizations were typically small and cannot account for the robust biases found in the Categorization experiments. Moreover, the above mentioned simpler measures lead to the same pattern of results.

***Adaptation.*** Psychometric functions based on a cumulative Gaussian were fitted to estimate each observer’s PSE of facial expression and their sensitivity (as indexed by the slope parameter) using Matlab R2015a with the Palamedes toolbox (Kingdom & Prins, 2009). Lapse rate was fixed at 0.03 and guess rate at 0. Goodness of fit was estimated with 400 simulations. If this analysis was significant (p < 0.05), deviance was deemed too large to be acceptable. Given that the PSE in the unadapted state might differ between different observers, we calculated the difference scores in PSE between adapted conditions and baseline to analyze and present the data.

### 3.2 Results

**Fig. 3:**
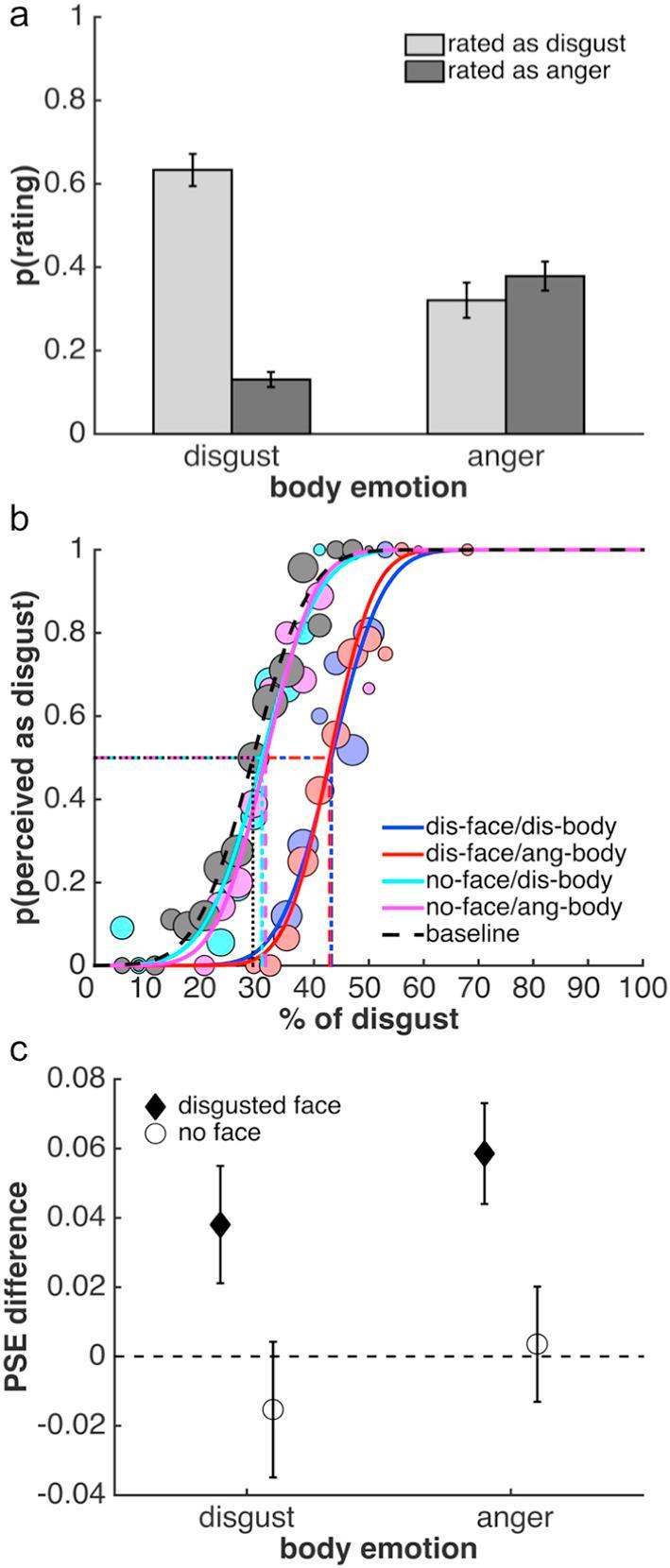
Results of Study 1. Facial expressions in this study always showed disgust. (a) Mean proportion of perceptual categorizations as disgusted or angry in response to a disgusted facial expression in a disgusted or angry body context. (b) Psychometric functions from one example observer to illustrate the results of the adaptation task. Dotted lines indicate PSEs, the size of the circles illustrates the number of trials for a given morph level. As can be seen, there is no difference between the baseline PSE and PSEs after adaptation to body contexts alone. Adaptation to a disgusted face generates aftereffects, as indicated by the difference of the respective PSEs to baseline. These aftereffects are independent of body context and consistent with adaptation to disgust. (c) Facial expression aftereffects plotted as the mean difference between the baseline PSE and PSEs after adaptation to face-body stimuli, for a disgusted facial expression with a disgusted or angry body (diamonds), and for the two body contexts without faces (circles). Error bars denote ±1 SEM in all plots.

***Perceptual Categorization.*** The full confusion matrix is presented in Table 1. For the current study, the effects of disgusted and angry body contexts are critical and are the focus of the remainder of the analysis (Fig. 3a). These two body contexts had different effects on categorization: disgusted facial expressions were more often accurately categorized as disgust when presented in a disgusted than an angry body context (Fig. 3a; paired *t* test: *t*(22) = 7.37, *p* < 0.0001, *Hedges’ g* = 1.58). Conversely, they were judged more often to be angry when presented in an angry compared to a disgusted context (*t*(22) = −6.30, *p* < 0.0001, *Hedges’ g* = −1.84). Both of these effects indicate that presenting disgusted facial expressions in an incongruent angry body context leads to a perceptual bias away from disgust and towards anger. In contrast to previous studies (Aviezer et al., 2008, Noh and Isaacowitz, 2013), however, this effect did not lead to a full reversal of perceptual judgements: disgusted facial expressions were more often perceived as disgusted in a disgust context than they were perceived as angry in an anger context (*t*(22) = 5.19, *p* < 0.0001, *Hedges’ g* = 1.42). Similarly, disgusted faces were less often judged as angry in a disgust context compared to how often they were judged as disgusted in an angry context (*t*(22) = −3.86, *p* < 0.001, *Hedges’ g* = −1.20).

**Table 1:** Confusion matrix of Experiment 1, showing the mean proportion ±1 SEM of categorisations of disgusted facial expressions in different body contexts. The shaded cells represent the influence of body context on perception of facial expressions, i.e., the proportion of trials, on which the face was reported to show the emotion of the body.

***Adaptation.*** Adaptation was only affected by the physical properties of the disgusted facial expression; the body context had no influence on adaptation either in conjunction with the facial expression or on its own (Fig. 3b and c). A 2 × 2 repeated measures ANOVA (disgust facial expression [present versus absent] × body posture [disgusted versus angry]) indicated a main effect of whether a face was present (*F*(1,20) = 9.79, *p* < 0.01), but no other main effect or interaction (Fig. 3b and c). Adaptation led to an aftereffect both when a face was shown in a disgust context (one-sample *t* test: *t*(20) = 2.25, *p* < 0.05, *g_1_* = 0.49) or an anger context (*t*(20) = 4.03, *p* < 0.001, *g_1_* = 0.88). Importantly, and as already indicated by the ANOVA, these aftereffects, which were consistent with adaptation to disgust, did not differ between conditions (*t*(20) = −1.31, *p* > 0.2). Adaptation to a disgusted body without a face (*t*(20) = −0.78, *p* > 0.4), or an angry body without a face (*t*(20) = 0.21, *p* > 0.8), did not lead to any measurable aftereffects. Again, as indicated by the ANOVA, there was no difference between these two conditions (*t*(20) = −0.79, *p* > 0.4). There were no significant main effects or interactions in the analysis of the slope values of the psychometric functions.

We conducted a directional Bayesian paired-sample *t* test with default prior distribution to evaluate evidence for the absence of a difference between the aftereffects after adaptation to disgusted faces in disgust and anger body contexts. This additional analysis indicated that the null hypothesis of no difference in aftereffects was preferred to the alternative by a Bayes factor of 9.12. Such a value is typically considered to provide substantial evidence (Jeffreys, 1961). A caveat is that it is difficult to estimate the effect size, which would determine the prior for this analysis. Importantly, however, a Bayes factor robustness check indicated that the data provided substantial evidence for the absence of a difference in aftereffects over a reasonably large range, down to a prior width of approximately 0.2. It is important to highlight that the Bayes factor analysis means that our data provide positive evidence for the absence of an effect, rather than suffering from an absence of evidence for an effect, which is the typical concern with null results.

***Relationship between experiments.*** Supporting the evidence for differential influences of body context on categorization and adaptation was the absence of a relationship between these effects on a subject-by-subject basis: the extent of the influence of body context on categorization, as measured by both Bias Indices, was unrelated to the difference in aftereffects in the two body contexts (Bias Index 1: *Pearson’s r* = −0.12, *p* > 0.55; Bias Index 2: *Pearson’s r* = −0.10, *p* > 0.60).

### 3.3 Discussion

In keeping with previous research, Study 1 shows that perceptual categorizations of facial expressions are biased by body posture (Hassin et al., 2013). Importantly for our question, a disgusted facial expression with an angry body is more likely to be judged as angry compared to when it is viewed with a disgusted body. Yet, adaptation was not affected by body context: adaptation to disgusted expressions, in a disgusted or angry body, resulted in aftereffects with a similar PSE. Additionally, there was no relationship between the size of the categorization bias and the difference in aftereffects. This finding indicates that categorization and adaptation might target different visual representations: while aftereffects are based on representations of facial representations that have not yet incorporated information from body context, categorization is based on the integrated face-body percept.

One caveat of this conclusion, however, is the fact that our biasing effect of body context is weaker than reported in some (Aviezer et al., 2008, Aviezer et al., 2012, Noh and Isaacowitz, 2013), but not all, previous studies (e.g., (Meeren et al., 2005). Note, that those previous studies showing strong effects used identical stimuli, suggesting that the size of the effect may be related to specific stimulus characteristics. Nevertheless, one could argue that the lack of a difference in the two adaptation conditions here might be due to the fact that the perceptual bias induced by body context was too small. We explored this possibility in Study 2.

## 4 Study 2: Even strong influences of body context on perceptual categorization do not affect adaptation

Here, we chose a subset of compound face-body stimuli so as to increase the influence of the angry body context on disgusted faces.

### 4.1 Method

#### 4.1.1 Observers

A new set of 24 observers (5 male; age in years (mean ± std): 21.4 ± 1.7) participated in Study 2. One observer had to be excluded from both experiments because they discontinued to participate after the first visit. In the adaptation task, three participants were excluded from the analysis because of a poor fit between data and psychometric function in one or more conditions as indicated by the goodness-of-fit analysis.

#### 4.1.2 Stimuli

Based on the data from the categorization experiment in Study 1, we selected the top third compound face-body stimuli showing the highest body context bias (i.e. where the disgusted face was rated most often as angry when presented in an angry body context). Only these eight identities were used as face-body stimuli in Study 2. Target stimuli in the Adaptation experiment were the same as before.

#### 4.1.3 Procedure and data analysis

The procedure of Study 2 was identical to Study 1 except for two aspects: First, given that we used eight rather than 24 different identities, each session of the Perceptual Categorization experiment consisted of 32 trials. Second, given that Study 1 showed that adaptation to the body context on its own has no effect on subsequent perception of facial expressions, we did not include adaptation to body context without a face. Data analysis was identical to that used for Study 1.

### 4.2 Results

**Fig. 4:**
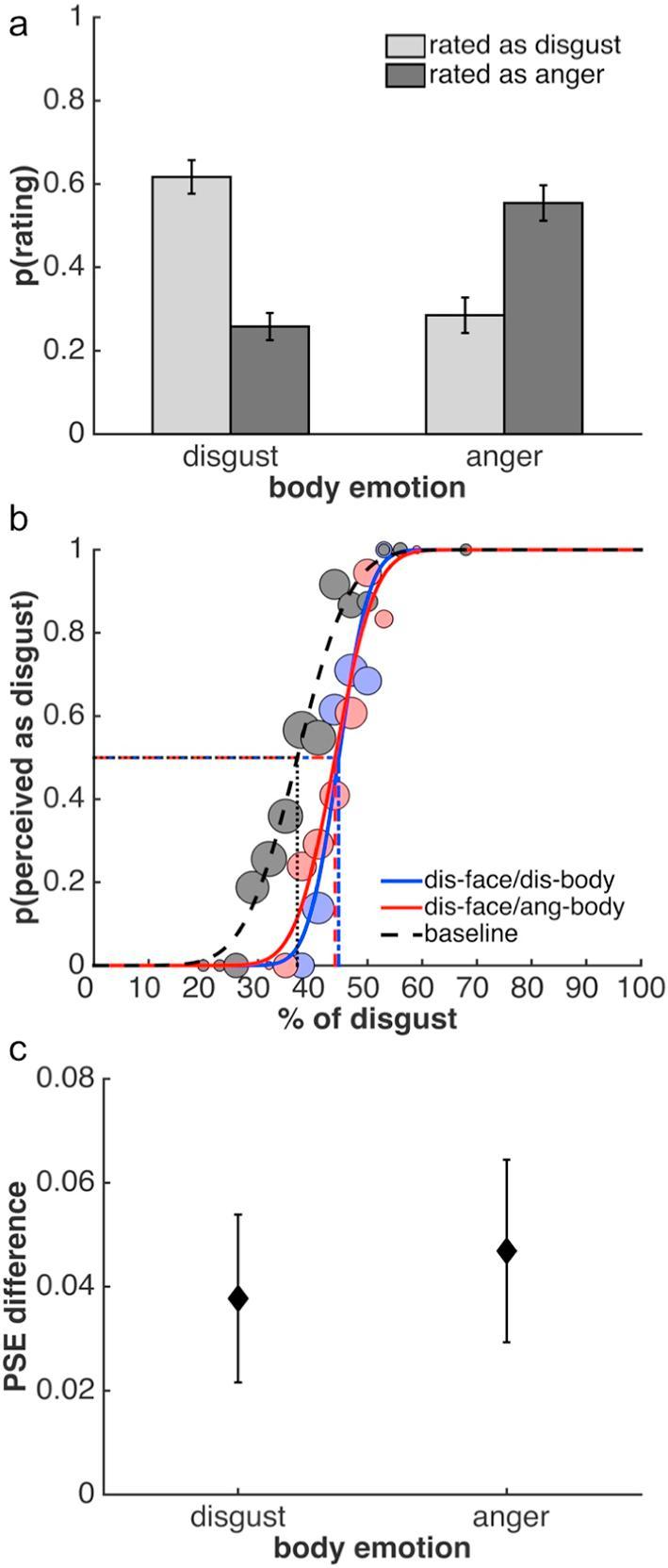
Results of Study 2. Facial expressions in this study always showed disgust. (a) Mean proportion of perceptual categorizations as disgusted or angry in response to a disgusted facial expression in a disgusted or angry body context. (b) Psychometric functions from one example observer to illustrate the results of the adaptation task. Adaptation to a disgusted face generates aftereffects, which are independent of body context and consistent with adaptation to disgust. (c) Facial expression aftereffects plotted as the mean difference between the baseline PSE and PSEs after adaptation to face-body stimuli with a disgusted facial expression and a disgusted or angry body. Further details as in Fig. 3.

***Perceptual Categorization.*** Table 2 shows the full confusion matrix. Similar to Study 1, we were primarily interested in the effects of disgusted and angry body contexts and the analysis focuses on these conditions (Fig. 4a). Mirroring the findings of Study 1, viewing disgusted faces in the context of a disgusted or angry body posture had different effects on how observers categorized the facial expressions: Disgusted facial expressions were more often correctly categorized as disgusted when presented in a disgusted than an angry body context (Fig. 4a; paired *t* test: *t*(22) = 5.62, *p* < 0.0001, *Hedges’ g* = 1.64). Conversely, these same expressions were judged more often to be angry when presented in an angry compared to a disgusted context (*t*(22) = −5.24, *p* < 0.0001, *Hedges’ g* = −1.60). Importantly, as intended through our stimulus selection process, the body context induced a full reversal of perceptual categorizations: there was no difference in how often disgusted facial expressions were perceived as disgusted in a disgust context compared to how often they were perceived as angry in an anger context (*t*(22) = 1.31, *p* > 0.2). Similarly, there was no difference in how often disgusted expressions were perceived as angry in the disgust context compared to how often they were perceived as disgusted in an anger context (*t*(22) = −0.58, *p* > 0.55).

**Table 2:** Confusion matrix of Study 2, showing the mean proportion ±1 SEM of categorisations of disgusted facial expressions in different body contexts. Further details as in Table 1.

***Adaptation.*** Results from the adaptation tasks (Fig. 4b and c) suggest a similar conclusion to that of Study 1: adaptation was not affected by body context. Adaptation induced an aftereffect when a face was shown in both the disgust context (one-sample *t* test: *t*(19) = 2.34, *p* < 0.05, *g_1_* = 0.52) and the anger context (*t*(19) = 2.67, p < 0.05, *g_1_* = 0.60). However, again the aftereffects were consistent with adaptation to disgust and did not differ between conditions (paired-sample *t* test; *t*(19) = −0.4352, *p* > 0.65). A Bayes factor analysis indicated that the null hypothesis of no difference was preferred to the alternative by a Bayes factor of 5.78 (directional Bayesian paired-sample *t* test). A Bayes factor robustness check indicated that the data provided substantial evidence for the absence of a difference in aftereffects over a reasonably large range, down to a prior width of approximately 0.35. Slope values of the psychometric functions did not differ between conditions.

***Relationship between experiments.*** As in Study 1, the influence of body context as measured by both Bias Indices was not related to the difference in aftereffects in the two body contexts (Bias Index 1: *Pearson’s r* = −0.06, *p* > 0.80; Bias Index 2: *Pearson’s r* = −0.11, *p* > 0.60), again supporting a dissociation between the two tasks.

### 4.3 Discussion

Study 2 indicates that, even when using stimuli that lead to a strong influence of angry body context on categorization of disgusted facial expressions, body context has no effect on adaptation. This supports the conclusion from Study 1 that categorization and adaptation tap into different face representations.

In Studies 1 and 2 we used a design, in which categorization and adaptation were measured in different experiments. This approach has the advantage that each task is conducted in a paradigm-typical way and findings can easily be compared to results reported in the literature. However, the following criticism could be levelled against this approach: while body context clearly induces a biasing effect on perception of facial expressions in the categorization task, it is possible that the specific setting of the adaptation task meant that body context did not induce a bias in the face-body stimuli used as adaptor. If this were the case, it would not be surprising that body context does not influence adaptation. Despite the fact that this scenario seems unlikely *a priori*, we addressed this potential issue in Study 3.

## 5 Study 3: No effect of body context on adaptation despite measuring strong influences of body within the adaptation paradigm

In order to test the hypothesis that body context does not induce a perceptual bias in the adaptation stimuli, we directly measured perceptual categorization of the adaptation stimuli. Categorization and adaptation were thus measured in the same experiment.

### 5.1 Method

#### 5.1.1 Observers

A new set of 12 observers (3 male; age in years (mean ± std): 19.4 ± 1.1) participated in Study 3. In this study, the categorization response had to be performed under time constraints and directly had to be followed by a second response to the target stimuli of the adaptation task. Some observers found this difficult. As a consequence, two observers had a high number of no-response trials in the Categorization task (>20%) and were close to chance performance in the remaining trials. These observers were excluded from further analysis of the Categorization task.

#### 5.1.2 Stimuli

Adaptation and target stimuli were identical to those used in Study 2 in the Adaptation experiment.

#### 5.1.3 Procedure and data analysis

**Fig. 5:**
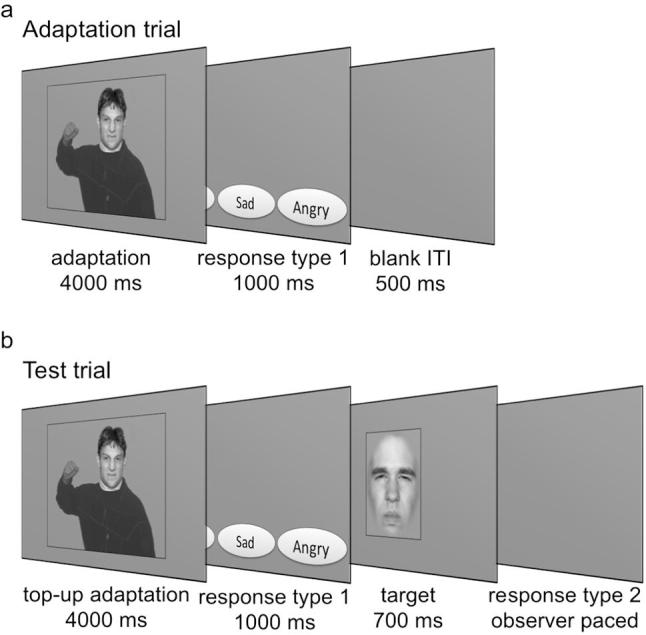
Illustration of the experimental designs of Study 3. (a) During adaptation observers were adapted to one of two face-body combinations: a disgusted face was shown in an angry body context – as illustrated here – or a disgusted context; after presentation of an adaptation stimulus, observers were asked to indicate the facial expression by clicking on one of four buttons with the mouse (buttons are not to size). (b) On test trials, observers were shown a top-up adaptation stimulus and were asked to indicate facial expression by clicking on one of four buttons with the mouse. This was followed by a target facial expression (morph between disgusted to angry). Again, observers were asked to indicate the facial expression, this time by pressing one of two buttons on the response box. As before, all indicated timings include a sinusoidally modulated ramp into (100 ms) and out of the full image (100 ms). This was used to minimize retinal aftereffects.

The procedure of Study 3 differed from that of the previous studies in a few key aspects because we did not conduct separate categorization and adaptation experiments. Rather, during the adaptation task, observers were asked to categorize each adaptation stimulus by choosing one of four buttons for disgust, fear, sadness, and anger (similar to how responses were collected in the categorization task in the previous studies) (Fig. 5). Subsequently, they had to respond to the target stimulus.

Observers participated in a congruent and an incongruent condition, with disgusted faces shown either in a disgust or anger body context, respectively. Conditions were counterbalanced across observers and conducted on a single visit with a break of 10 min between conditions to wash out adaptation. Before the first and after the second condition, observers participated in a baseline task that was identical to that in previous studies. The general data analysis approach was identical to that used before.

### 5.2 Results

**Fig. 6:**
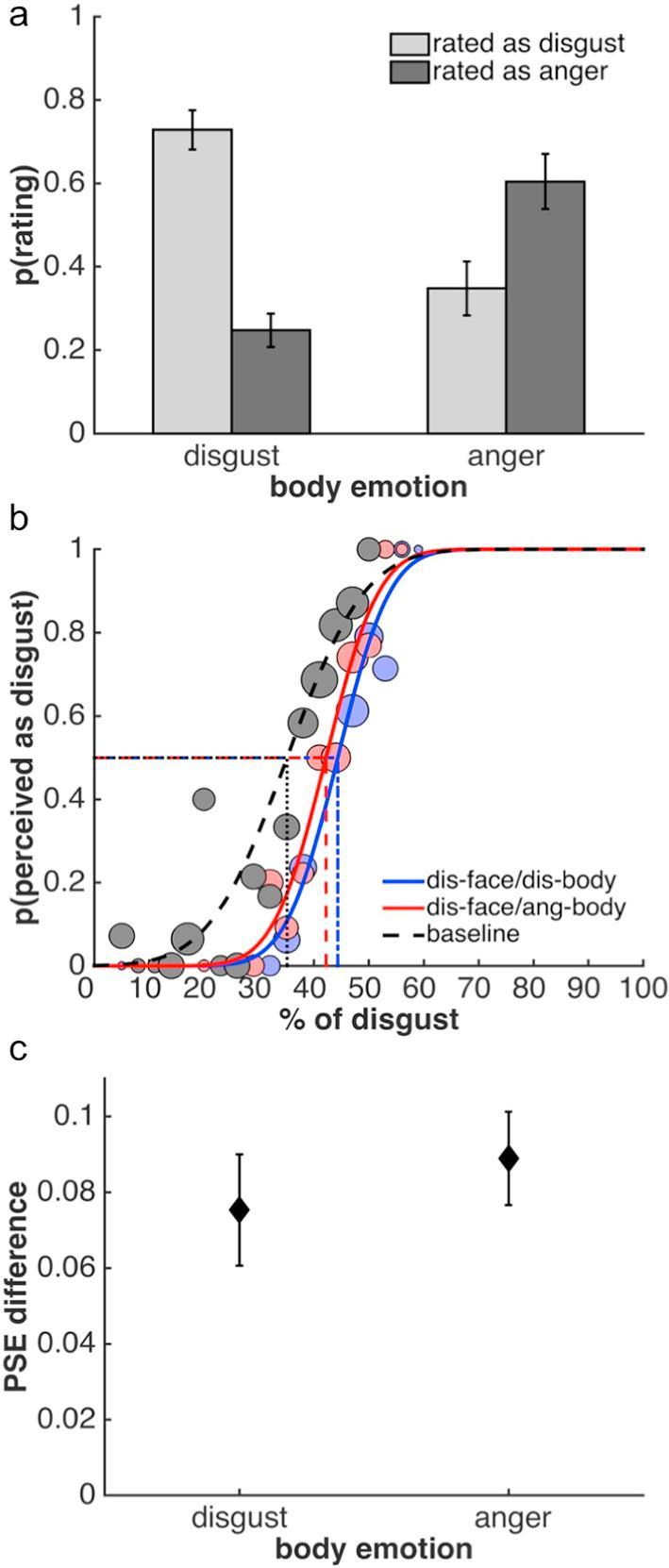
Results of Study 3. Facial expressions in this study always showed disgust. (a) Mean proportion of perceptual categorizations as disgusted or angry in response to a disgusted facial expression in a disgusted or angry body context, presented during adaptation. (b) Psychometric functions from one example observer to illustrate the aftereffects induced by adaptation. Adaptation to a disgusted face generates aftereffects, which are independent of body context and consistent with adaptation to disgust. (c) Facial expression aftereffects plotted as the mean difference between the baseline PSE and PSEs after adaptation to face-body stimuli with a disgusted facial expression and a disgusted or angry body. Further details as in Fig. 3.

For the full confusion matrix, please see Table 3. Similar to the previous two studies, viewing disgusted faces in the context of a disgusted or angry body posture affected categorization of facial expressions (Fig. 6a): Observers categorized disgusted facial expressions more often correctly as disgusted when presented in a disgusted than an angry body context (Fig. 6a; paired *t* test: *t*(9) = 4.32, *p* < 0.01, *Hedges’ g* = 2.03). Furthermore, disgusted expressions were judged more often to be angry when presented in an angry compared to a disgusted context (*t*(9) = −3.90, *p* < 0.01, *Hedges’ g* = −1.97). Similar to Study 2, the number of times disgusted facial expressions were perceived as disgusted in a disgust context was not significantly different from how often they were perceived as angry in an anger context (*t*(9) = 1.93, *p* = 0.09). Similarly, the number of times disgusted expressions were perceived as angry in the disgust context was not significantly different from to how often they were perceived as disgusted in an anger context (*t*(9) = −1.55, *p* > 0.15). In both instances, however, there may be a trend towards a difference

**Table 3:** Confusion matrix of Study 3, showing the mean proportion ±1 SEM of categorisations of disgusted facial expressions in different body contexts. Further details as in Table 1.

Despite the fact that we found clear evidence that perception of the adaptation stimuli is biased by body context, as in the previous two studies, the aftereffects suggest that adaptation was not affected by body context (Fig. 6b and c). We found robust aftereffects when a disgusted face was shown in both the disgust context (one-sample *t* test: *t*(11) = 5.13, *p* < 0.001, *g_1_* = 1.48) and the anger context (*t*(11) = 7.22, p < 0.001, *g_1_* = 2.09). Importantly, however, in both contexts, aftereffects were consistent with adaptation to disgust and did not differ between contexts (paired-sample *t* test; *t*(11) = −1.21, *p* > 0.25). The null hypothesis of no difference was preferred to the alternative by a Bayes factor of 6.69 (directional Bayesian paired-sample *t* test). A robustness check indicated that the data provided substantial evidence for the absence of a difference in aftereffects over a reasonably large range, down to a prior width of approximately 0.25. Slope values of the psychometric functions did not differ between conditions.

Despite the smaller sample size, Study 3 supported the conclusions from Studies 1 and 2: the influence of body context on categorization (as measured by both Bias Indices) was not related to the difference in aftereffects in the two body contexts (Bias Index 1: *Pearson’s r* = −0.11, *p* > 0.75; Bias Index 2: *Pearson’s r* = −0.06, *p* > 0.85), even with categorization and adaptation measured within the same experiment.

### 5.3 Discussion

In Study 3, we measured the influence of body context on categorization during adaptation. In other words, the two distinct experiments of Studies 1 and 2 were combined into one experiment. This design is unusual when compared to previous work studying both perceptual categorization of and adaptation to facial expressions. Importantly, however, it allows us to directly address whether the lack of influence of body posture on adaptation in Studies 1 and 2, is due to the fact that body context did not induce a perceptual bias when viewing the adaptation stimuli. Study 3 provides clear evidence to rule out this possibility. In fact, despite substantial differences in the design of Study 3, the biasing effect of body context on categorization of facial expressions was similar to that of Study 2. At the same time, the disgusted faces that were categorized differently depending on whether they were shown in the disgust or anger context induced very similar aftereffects consistent with adaptation to disgust. If anything, these aftereffects were on average even larger than those found in the previous studies.

## 6 Study 4: Facial expressions influence adaptation

The aim of this study was to show that adaptation to angry facial expressions in congruent and incongruent body contexts would induce aftereffects in the opposite direction to the previous studies. Furthermore, we were interested in relating these opposing aftereffects and accompanying perceptual categorizations to those measured in previous experiments. For the sake of comparability, and because of the unusual design of Study 3, we reverted back to separate experiments to measure categorization and adaptation, similar to the design of Studies 1 and 2.

### 6.1 Method

#### 6.1.1 Observers

A new set of 24 observers (2 male; age in years (mean ± std): 20.9 ± 1.7) participated in Study 4. One participant discontinued participation after the first visit. Moreover, two participants had to be excluded from both tasks. Both observers had at least one poorly fitting psychometric function in the Adaptation task and a flat response profile in the Categorization task, i.e., responses around chance in all four contexts.

#### 6.1.2 Stimuli

Compound face-body stimuli had angry rather than disgusted facial expressions (Fig. 1b). All other stimuli were as previously.

#### 6.1.3 Procedure and data analysis

The procedure of Study 4 was identical to Study 1 except for two aspects: First, rather than using disgusted facial expressions for the compound face-body stimuli, angry faces were shown. Second, as in Study 2, we did not adapt observers to body context without a face. The data analysis strategy was identical to that used before.

### 6.2 Results

**Fig. 7:**
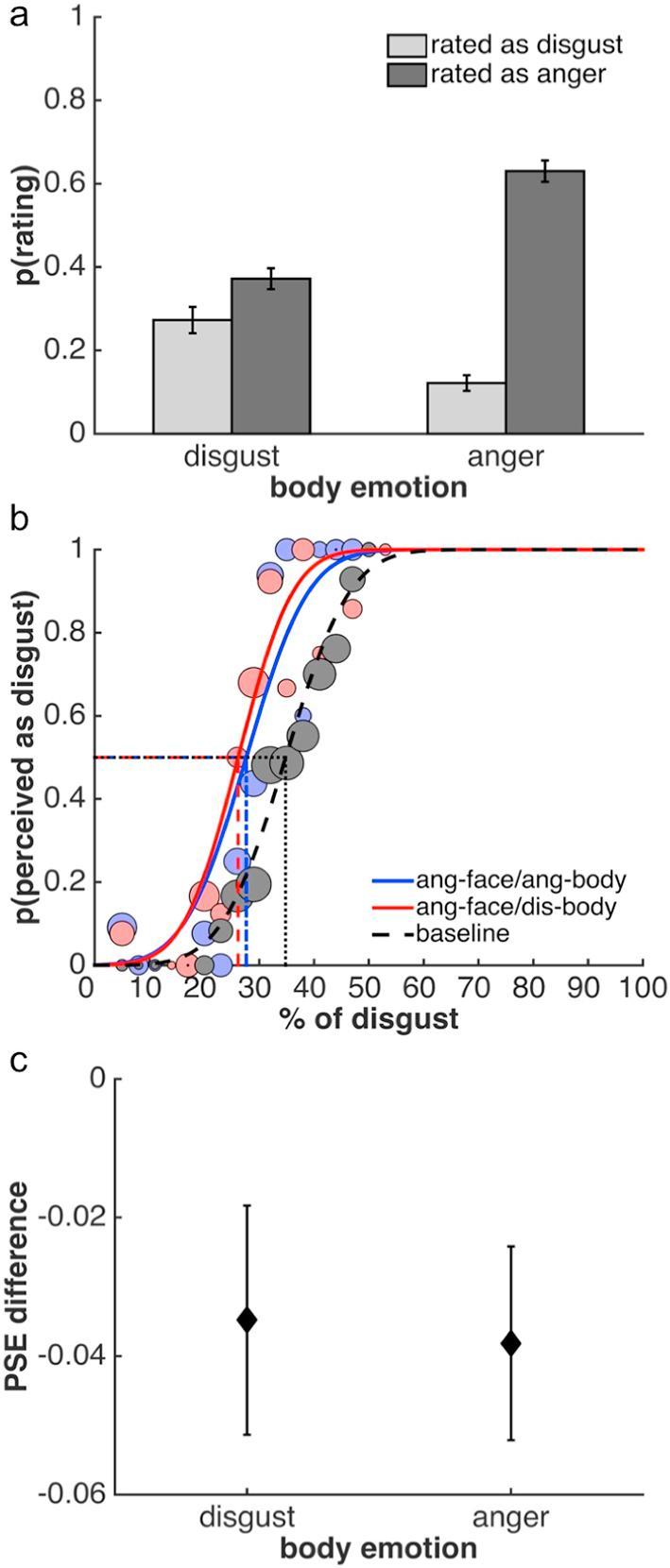
Results of Study 4. Facial expressions in this study always showed anger. (a) Mean proportion of perceptual categorizations as disgusted or angry in response to an angry facial expression in a disgusted or angry body context. (b) Psychometric functions from one example observer to illustrate the results of the adaptation task. Adaptation to an angry face generates aftereffects, which are independent of body context and consistent with adaptation to anger. (c) Facial expression aftereffects plotted as the mean difference between the baseline PSE and PSEs after adaptation to face-body stimuli with an angry facial expression and a disgusted or angry body. Further details as in Fig. 3.

***Perceptual Categorization.*** Table 4 shows the full confusion matrix. As with the previous studies, we were primarily interested in the effects of disgusted and angry body contexts and the analysis focuses on these conditions (Fig. 7a). As expected, viewing angry faces in the context of an angry or disgusted body posture had different effects on observers’ categorization.

**Table 4:** Confusion matrix of Study 4, showing the mean proportion ±1 SEM of categorisations of angry facial expressions in different body contexts. Further details as in Table 1.

Angry facial expressions were more often correctly categorized when presented in an angry than a disgusted body context (Fig. 7a; paired *t* test: *t*(20) = −6.78, *p* < 0.0001, *Hedges’ g* = −2.19). Angry faces were judged more often to be disgusted when presented in a disgusted compared to an angry context (*t*(20) = 4.10, *p* < 0.001, *Hedges’ g* = 1.24). These findings demonstrate that presenting an angry face in a disgusted body context induces a perceptual bias away from anger and towards disgust. Similar to Study 1, however, this effect did not lead to a full reversal of perceptual categorizations: in the anger context, angry facial expressions were more often perceived as angry than they were perceived as disgusted in the disgust context (*t*(20) = −11.55, *p* < 0.0001, *Hedges’ g* = −2.67). Furthermore, in the anger context, expressions were less often judged as disgusted compared to how often they were judged as angry in the disgust context (*t*(20) = 8.54, *p* < 0.0001, *Hedges’ g* = 2.43).

***Adaptation.*** Again, adaptation was not affected by body context (Fig. 7b and c). Aftereffects were found in both the anger context (one-sample *t* test: *t*(20) = −2.73, *p* < 0.05, *g_1_* = −0.60) and the disgust context (*t*(20) = −2.11, p < 0.05, *g_1_* = 0.46). Importantly, the aftereffects did not differ between conditions (*t*(20) = −0.21, *p* > 0.8) and were consistent with adaptation to anger, i.e., the PSE was biased away from the anger adaptor and thus in the opposite direction to those in the previous two studies. Again, a Bayes factor analysis indicated that the null hypothesis of no difference was preferred to the alternative (Bayes factor 5.10; directional Bayesian paired-sample *t* test). A Bayes factor robustness check indicated that the data provided substantial evidence for the absence of a difference in aftereffects down to a prior width of approximately 0.4. Slope values of the psychometric function did not differ between conditions.

***Relationship between experiments.*** Again, the influence of body context as measured by both Bias Indices was not related to the difference in aftereffects in the two body contexts (Bias Index 1: *Pearson’s r* = −0.06, *p* > 0.75; Bias Index 2: *Pearson’s r* = −0.05, *p* > 0.80), providing support for a dissociation between the two experiments.

***Overall pattern of results across Studies 1 to 4.*** Certain aspects of the results generated by the four studies show a pattern that approaches a double dissociation. This is most clearly illustrated by comparing results of Studies 2 and 4. In Study 2, a clear difference in categorization is accompanied by a lack of difference in aftereffects. At the same time, opposing aftereffects across Studies 2 and 4 in the angry context condition are not accompanied by a similarly clear difference in categorizations. This pattern of results suggests that the lack of an influence of body context on adaptation is not due to a lack of power or, more generally, the overall size of the biasing effect of body on categorization.

***Global analysis across Studies 1 to 4.*** Essentially, all four studies assessed the same question, and in a final analysis, we collapsed the data across all studies. As in all previous analyses, in the Categorization experiments, only disgust and anger responses as well as disgust and anger contexts were considered. All responses were re-coded as correct or incorrect, and contexts were re-coded as congruent or incongruent with the facial expression. Note that correct and incorrect responses are not dependent. In the adaptation experiments, deviations of PSE estimates from baseline were re-coded such that aftereffects in the direction consistent with adaptation to the facial expression resulted in positive and, those in the opposite direction, in negative values.

As expected, viewing facial expressions in the context of congruent or incongruent body contexts had robust effects on the observers’ categorization. Facial expressions were more often correctly categorized when presented in a congruent than an incongruent context (paired *t* test: *t*(76) = 11.80, *p* < 0.0001, *Hedges’ g* = 1.77), and less often incorrectly categorized (paired *t* test: *t*(76) = −9.45, *p* < 0.0001, *Hedges’ g* = −1.39). Not surprisingly, these findings support previous conclusions that body context can induce a perceptual bias towards the emotion expressed by the posture. However, across studies, this effect is not strong enough to lead to a full reversal of perceptual categorizations: facial expressions were more often correctly identified in the congruent context, than they were incorrectly categorized in the incongruent context (*t*(76) = 7.76, *p* < 0.0001, *Hedges’ g* = 1.07). Moreover, expressions were less often incorrectly categorized in the congruent context than they were correctly identified in the incongruent context (*t*(76) = −5.82, *p* < 0.0001, *Hedges’ g* = −0.91).

Across all studies, adaptation was not affected by body context. While robust aftereffects were found in both congruent (one-sample *t* test: *t*(73) = 5.15, *p* < 0.0001, *g_1_* = 0.60) and incongruent contexts (*t*(73) = 6.98, p < 0.0001, *g_1_* = 0.81), these aftereffects did not differ between contexts (*t*(73) = −1.33, *p* > 0.15) and were consistent with adaptation to the facial expression. A Bayes factor analysis indicated that the null hypothesis of no difference was preferred to the alternative (Bayes factor 17.21; directional Bayesian paired-sample *t* test). A Bayes factor robustness check indicated that the data provided substantial evidence for the absence of a difference in aftereffects down to a prior width of approximately 0.1.

Across all studies, we found support for a dissociation between categorization and adaptation: the influence of body context as measured by both Bias Indices was not related to the difference in aftereffects in the two contexts (Bias Index 1: *Pearson’s r* = −0.03, *p* > 0.75; Bias Index 2: *Pearson’s r* = −0.10, *p* > 0.40). The lack of a relationship was supported with substantial evidence by an analysis using Bayesian Pearson correlations with the standard Beta prior width of 1 (Bias Index 1: *Pearson’s r* = −0.03, Bayes factor in favour of the null 6.08; Bias Index 2: *Pearson’s r* = −0.10, Bayes factor 4.77).

## 7 General discussion

Consistent with previous research, we show that viewing facial expressions within an incongruent body context biases perceptual categorization of the expression towards the body emotion. Critically, however, we also demonstrate that adaptation to facial expressions is unaffected by body context. Moreover, there is no relationship between the size of the biasing effect of body context on categorization and adaptation-induced aftereffects. Our results suggest that adaptation to, and categorization of, facial expressions are based on different types of face representations: While aftereffects arise from representations of facial expressions that are not integrated with information about body posture, performance in the Categorization Task is based on integrated whole-person representations. We therefore conclude that the integration of face and body information occurs downstream of the sites of adaptation. The results enhance our understanding of functional organisation of social perception and conflict with previous claims of an early integration of face and body information. Furthermore, the findings provide insights into the functional role of adaptation to high-level stimulus properties.

In everyday life, we typically interact with whole agents rather than isolated faces, and a person’s facial expressions and body postures represent a single set of underlying emotions. Using integrated whole-person information therefore can provide a unified cue to understanding other people’s emotion in everyday social interactions. This might be the reason why, for instance, poker players scrutinize both the face and body of opponents for clues about their feelings and intentions (Hayano, 1980). Despite this importance, we know little about how the brain processes and codes whole-person representations. The results of the current study offer some insight into how such representations arise, specifically with respect to emotional signals. In particular, they provide evidence for a specific hierarchical organisation of whole-person perception: while visual mechanisms at different levels of the visual information-processing hierarchy contribute to adaptation to facial expression (Xu et al., 2008), our adaptation procedure included manipulations to ensure that we target high-level facial expression representations, minimising the contribution of adaptation to low-level features. The finding that these high-level representations are not affected by body context suggests that emotional information from face and body is initially processed in largely parallel and independent streams; integrated whole-person representations are generated after the points in the information-processing hierarchy, at which adaptation to facial expression takes place.

This functional interpretation of our results is supported by our current understanding of the neuronal architecture of face processing and of adaptation to facial characteristics. While there is good evidence that, functionally, high-level face aftereffects such as those studied here are the result of a re-calibration process (Webster & MacLeod, 2011), the underlying neuronal mechanisms are less well understood; in fact, most likely, several different processes could contribute to adaptation at the neuronal level (Grill-Spector et al., 2006). Importantly, these effects may be shaped by top-down processing within a network of face-sensitive areas (Ewbank, Henson, Rowe, Stoyanova, & Calder, 2013), suggesting that adaptation mechanisms may not be limited to a single anatomical site. Independently of the precise neuronal mechanisms underlying aftereffects, however, there is substantial neuroimaging evidence that facial expression representations, as targeted by our adaptation procedure, are underpinned by neuronal populations in regions of the core face perception system, including STS and FFA (e.g., Fox et al., 2009, Furl et al., 2007, Furl et al., 2007, Ganel et al., 2005, Winston et al., 2004). Our finding that such facial expression representations are insensitive to body context thus suggests that integrated whole-person representations arise downstream from these core face perception areas. This finding is in line with a small but growing literature from monkey electrophysiology and monkey/human neuroimaging. Here, studies have identified parallel and largely separate networks for faces and bodies along occipital and temporal cortices, with integration happening only in anterior temporal lobes (Fisher and Freiwald, 2015, Harry et al., 2016, Premereur et al., 2016). Our findings extend such a hierarchical model of social perception, demonstrating that signals coding a person’s emotional state follow similar processing principles as those for non-emotional faces and bodies.

Our results are at odds, however, with previous suggestions of early integration of facial expression and body context in extrastriate areas of occipital cortex. Three sets of results have been invoked to support the early integration hypothesis. First, disgusted faces in an angry body context elicit eye-movement scan-paths characteristic of isolated angry facial expressions, and vice versa (Aviezer et al., 2008). This finding is equally, if not more, consistent with a modulatory influence on pre-perceptual sampling rather than on perceptual integration per se. Second, the integration of facial expression and body posture information is unaffected by an observer’s intention or cognitive load and has thus been argued to be automatic (Aviezer et al., 2011). This automaticity, however, in itself, does not speak to the nature of these processes, i.e., whether they are early, late, perceptual, pre-perceptual, or post-perceptual. Finally, the event-related potential component P1, which is thought to originate from lateral and dorsal parts of the occipital cortex, is enhanced when observers view an incongruent face-body stimulus in comparison to a congruent stimulus (Meeren et al., 2005). However, as the authors of the Meeren study point out (Meeren et al., 2005), the enhancement of the P1 component could reflect early incongruency-detection rather than genuine integration of perceptual information. Interpreted thus, work supporting initially parallel streams of social information processing are easily reconciled with Meeren and colleagues’ study: early incongruency detection leaves independent featural analysis of face and body intact.

There is an alternative interpretation to the finding that body context has no influence on facial expression aftereffects, which would not necessarily be inconsistent with an early integration account. Specifically, if there were two or more parallel channels encoding facial expressions, information from facial expression and body posture could be combined at an early stage in one of these channels – accounting for the biasing effect of body context in the Categorization Task – but not in the other channel(s) – explaining the lack of a contextual influence on the aftereffects. However, such an interpretation makes a number of largely unsupported assumptions. First, there would have to be at least two parallel channels encoding aspects of facial expression. Potential candidate parallel channels might include STS and FFA, respectively, since both regions have been shown to encode aspects of facial expression (for recent review of a neural model of face processing, see Duchaine & Yovel, 2015). However, only one of these channels would have to be amenable to facial expression adaptation, an assumption, which is not supported for STS and FFA (e.g., Fox et al., 2009). Finally, perceptual decisions in the Categorization Task and in the Adaptation Task would have to be based on information from different channels, and it is not clear on what grounds such an assumption would be warranted. While these combined assumptions are not impossible, they seem unlikely given our current understanding of facial expression processing.

Our results also provide insight into the functional role of adaptation to high-level stimulus properties. They indicate that changes in neuronal sensitivity underlying aftereffects occur in response to isolated physical characteristics of a face rather than the integrated, global conscious percept. Results from psychophysics and monkey electrophysiology suggest that the brain represents facial characteristics, including expressions, as points within a face space centred on a neutral norm (e.g., Cook et al., 2011, Leopold et al., 2006, Leopold et al., 2001, Rhodes et al., 2017, Skinner and Benton, 2010, Webster and MacLeod, 2011). The average norm or origin of the coordinate system is continuously updated to optimally represent the faces an observer encounters, a re-calibration that underlies perceptual aftereffects. Our results suggest that the high-level representations amenable to adaptation are optimised to process the current diet of *physical* features of expressions rather than the perceptually experienced global *emotional* signals. This interpretation fits neatly with results from a previous neuroimaging study (Furl, van Rijsbergen, Treves, & Dolan, 2007a), demonstrating a dissociation between adaptation and conscious perception, and that processes underlying the perceptual experience of facial expressions are downstream from processes responding to adaptation. Considerations concerning the wider significance of this finding have to remain speculative at present. Nevertheless, our results may have implications for a wider understanding of how the brain generates integrated percepts of complex objects. In particular, the strategy of initially calibrating information processing with respect to individual components rather than the integrated percept, as suggested by our results for social stimuli, may extend to complex objects. This type of calibration might be useful in circumstances when individual components are used to form not just one but several, partly independent, integrated representations. For instance, faces and bodies might be integrated to form representations of a person’s identity and emotions. Calibration of information processing with respect to faces prior to the formation of an integrated face-body percept might be important to avoid cross-dimensional influences.

There is evidence suggesting that the influence of body context on facial categorization is a genuine perceptual effect rather than being due to post-perceptual interpretative processes (Aviezer et al., 2012), and our findings do not challenge this idea. In agreement with previous work (Aviezer et al., 2008, Noh and Isaacowitz, 2013), observers did not ignore faces in favor of body context, neither were they more uncertain when faces were in an incongruent body context. If that were so, similar biases would be expected for all incongruent contexts, which was not the case.

In conclusion, we confirmed that body context biases perceptual categorization of facial expressions but show that these biases are not due to an early integration of visual information from face and body. In contrast to previous claims, even high-level representations of facial expressions encoded late in the visual system in temporal cortex are unaffected by body context. These findings are consistent with a hierarchical model of social perception, in which specialised mechanisms process signals from different social sources largely independently up to a high-level, configural stage before the output of their analysis is combined to generate the global, integrated representation underlying perceptual experience.

## Appendix A Supplementary material

Supplementary Data S1

This is open data under the CC BY license (http://creativecommons.org/licenses/by/4.0/).

Supplementary Data S2

This is open data under the CC BY license (http://creativecommons.org/licenses/by/4.0/).

Supplementary Data S3

This is open data under the CC BY license (http://creativecommons.org/licenses/by/4.0/).

Supplementary Data S4

This is open data under the CC BY license (http://creativecommons.org/licenses/by/4.0/).

Supplementary Data S5

This is open data under the CC BY license (http://creativecommons.org/licenses/by/4.0/).

Supplementary Data S6

This is open data under the CC BY license (http://creativecommons.org/licenses/by/4.0/).

Supplementary Data S7

This is open data under the CC BY license (http://creativecommons.org/licenses/by/4.0/).

Supplementary Data S8

This is open data under the CC BY license (http://creativecommons.org/licenses/by/4.0/).

## References

[b0005] Aviezer H., Bentin S., Dudarev V., Hassin R.R. (2011). The automaticity of emotional face-context integration. Emotion.

[b0010] Aviezer H., Hassin R.R., Ryan J., Grady C., Susskind J., Anderson A. (2008). Angry, disgusted, or afraid? Studies on the malleability of emotion perception. Psychological Science.

[b0015] Aviezer H., Trope Y., Todorov A. (2012). Holistic person processing: Faces with bodies tell the whole story. Journal of Personality and Social Psychology.

[b0020] Brainard D.H. (1997). The psychophysics toolbox. Spatial Vision.

[b0025] Bruce V., Young A. (2012). Face perception.

[b0030] Butler A., Oruç İ., Fox C.J., Barton J.J.S. (2008). Factors contributing to the adaptation aftereffects of facial expression. Brain Research.

[b0035] Cook R., Matei M., Johnston A. (2011). Exploring expression space: Adaptation to orthogonal and anti-expressions. Journal of Vision.

[b0040] de Gelder B., Meeren H.K.M., Righart R., Van den Stock J., van de Riet W.A.C., Tamietto M. (2006). Beyond the face: Exploring rapid influences of context on face processing. Progress in Brain Research.

[b0045] Duchaine B., Yovel G. (2015). A revised neural framework for face processing. Annual Review of Vision Science.

[b0050] Ewbank M.P., Henson R.N., Rowe J.B., Stoyanova R.S., Calder A.J. (2013). Different neural mechanisms within occipitotemporal cortex underlie repetition suppression across same and different-size faces. Cerebral Cortex.

[b0055] Fisher C., Freiwald W.A. (2015). Whole-agent selectivity within the macaque face-processing system. Proceedings of the National Academy of Sciences of the United States of America.

[b0060] Fox C.J., Barton J.J.S. (2007). What is adapted in face adaptation? The neural representations of expression in the human visual system. Brain Research.

[b0065] Fox C., Moon S., Iaria G., Barton J. (2009). The correlates of subjective perception of identity and expression in the face network: An fMRI adaptation study. NeuroImage.

[b0070] Furl N., van Rijsbergen N.J., Treves A., Dolan R.J. (2007). Face adaptation aftereffects reveal anterior medial temporal cortex role in high level category representation. NeuroImage.

[b0075] Furl N., van Rijsbergen N.J., Treves A., Friston K.J., Dolan R.J. (2007). Experience-dependent coding of facial expression in superior temporal sulcus. Proceedings of the National Academy of Sciences of the United States of America.

[b0080] Ganel T., Valyear K.F., Goshen-Gottstein Y., Goodale M.A. (2005). The involvement of the “fusiform face area” in processing facial expression. Neuropsychologia.

[b0085] Gilaie-Dotan S., Malach R. (2007). Sub-exemplar shape tuning in human face-related areas. Cerebral Cortex.

[b0090] Grill-Spector K., Henson R., Martin A. (2006). Repetition and the brain: Neural models of stimulus-specific effects. Trends in Cognitive Sciences.

[b0095] Harry B.B., Umla-Runge K., Lawrence A.D., Graham K.S., Downing P.E. (2016). Evidence for integrated visual face and body representations in the anterior temporal lobes. Journal of Cognitive Neuroscience.

[b0100] Hassin R.R., Aviezer H., Bentin S. (2013). Inherently ambiguous: Facial expressions of emotions, in context. Emotion Review.

[b0105] Haxby J.V., Hoffman E.A., Gobbini M.I. (2000). The distributed human neural system for face perception. Trends in Cognitive Sciences.

[b0110] Hayano D.M. (1980). Communicative competency among poker players. Journal of Communication.

[b0115] Hentschke H., Stüttgen M.C. (2011). Computation of measures of effect size for neuroscience data sets. European Journal of Neuroscience.

[b0120] Hsu S.M., Young A. (2004). Adaptation effects in facial expression recognition. Visual Cognition.

[b0125] Jeffreys H. (1961). The Theory of Probability.

[b0130] Kingdom F., Prins N. (2009). Psychophysics: A practical introduction.

[b0135] Kleiner M., Brainard D., Pelli D. (2007). What's new in psychtoolbox-3?. Perception.

[b0140] Kontsevich L.L., Tyler C.W. (1999). Bayesian adaptive estimation of psychometric slope and threshold. Vision Research.

[b0145] Langner O., Dotsch R., Bijlstra G., Wigboldus D.H.J., Hawk S.T., van Knippenberg A. (2010). Presentation and validation of the Radboud Faces Database. Cognition & Emotion.

[b0150] Leopold D.A., Bondar I.V., Giese M.A. (2006). Norm-based face encoding by single neurons in the monkey inferotemporal cortex. Nature.

[b0155] Leopold D.A., O'Toole A.J., Vetter T., Blanz V. (2001). Prototype-referenced shape encoding revealed by high-level aftereffects. Nature Neuroscience.

[b0160] Macmillan N.A., Creelman C.D. (2005). Detection theory: A user's guide.

[b0165] Meeren H., van Heijnsbergen C., de Gelder B. (2005). Rapid perceptual integration of facial expression and emotional body language. Proceedings of the National Academy of Sciences of the United States of America.

[b0170] Ng M., Ciaramitaro V.M., Anstis S., Boynton G.M., Fine I. (2006). Selectivity for the configural cues that identify the gender, ethnicity, and identity of faces in human cortex. Proceedings of the National Academy of Sciences of the United States of America.

[b0175] Noh S.R., Isaacowitz D.M. (2013). Emotional faces in context: Age differences in recognition accuracy and scanning patterns. Emotion.

[b0180] Premereur E., Taubert J., Janssen P., Vogels R., Vanduffel W. (2016). Effective connectivity reveals largely independent parallel networks of face and body patches. Current Biology.

[b0185] Quian Quiroga R., Kraskov A., Mormann F., Fried I., Koch C. (2014). Single-cell responses to face adaptation in the human medial temporal lobe. Neuron.

[b0190] Rhodes G., Pond S., Jeffery L., Benton C.P., Skinner A.L., Burton N. (2017). Aftereffects support opponent coding of expression. Journal of Experimental Psychology: Human Perception and Performance.

[b0195] Rotshtein P., Henson R.N.A., Treves A., Driver J., Dolan R.J. (2005). Morphing Marilyn into Maggie dissociates physical and identity face representations in the brain. Nature Neuroscience.

[b0200] Skinner A.L., Benton C.P. (2010). Anti-expression aftereffects reveal prototype-referenced coding of facial expressions. Psychological Science.

[b0205] Tottenham N., Tanaka J.W., Leon A.C., McCarry T., Nurse M., Hare T.A. (2009). The NimStim set of facial expressions: Judgments from untrained research participants. Psychiatry Research.

[b0210] Webster M.A., MacLeod D.I.A. (2011). Visual adaptation and face perception. Philosophical Transactions of the Royal Society B: Biological Sciences.

[b0215] Webster M.A., Kaping D., Mizokami Y., Duhamel P. (2004). Adaptation to natural facial categories. Nature.

[b0220] Winston J.S., Henson R.N.A., Fine-Goulden M.R., Dolan R.J. (2004). fMRI-adaptation reveals dissociable neural representations of identity and expression in face perception. Journal of Neurophysiology.

[b0225] Xu H., Dayan P., Lipkin R.M., Qian N. (2008). Adaptation across the cortical hierarchy: Low-level curve adaptation affects high-level facial-expression judgments. Journal of Neuroscience.

